# Estimated pulse wave velocity is associated with all-cause mortality and cardiovascular mortality among adults with diabetes

**DOI:** 10.3389/fcvm.2023.1157163

**Published:** 2023-04-17

**Authors:** Li-Da Wu, Peng Chu, Chao-Hua Kong, Yi Shi, Ming-Hui Zhu, Yi-Yuan Xia, Zheng Li, Jun-Xia Zhang, Shao-Liang Chen

**Affiliations:** Department of Cardiology, Nanjing First Hospital, Nanjing Medical University, Nanjing, China

**Keywords:** estimated pulse wave velocity, diabetes, arterial stiffness, NHANES, allcause mortality, cardiovascular mortality

## Abstract

**Aims:**

We aim to examine the association of estimated pulse wave velocity (ePWV) with all-cause and cardiovascular mortality in patients with diabetes.

**Methods:**

All of adult participants with diabetes from the National Health and Nutrition Examination Survey (NHANES) (1999–2018) were enrolled. ePWV was calculated according to the previously published equation based on age and mean blood pressure. The mortality information was obtained from the National Death Index database. Weighted Kaplan-Meier (KM) plot and weighted multivariable Cox regression was used to investigate the association of ePWV with all-cause and cardiovascular mortality risks. Restricted cubic spline was adopted to visualize the relationship between ePWV and mortality risks.

**Results:**

8,916 participants with diabetes were included in this study and the median follow-up duration was ten years. The mean age of study population was 59.0 ± 11.6 years, 51.3% of the participants were male, representing 27.4 million patients with diabetes in weighted analysis. The increment of ePWV was closely associated with increased risks of all-cause mortality (HR: 1.46, 95% CI: 1.42–1.51) and cardiovascular mortality (HR: 1.59, 95% CI: 1.50–1.68). After adjusting for cofounding factors, for every 1 m/s increase in ePWV, there was a 43% increased risk of all-cause mortality (HR: 1.43, 95% CI: 1.38–1.47) and 58% increased of cardiovascular mortality (HR: 1.58, 95% CI: 1.50–1.68). ePWV had positive linear associations with all-cause and cardiovascular mortality. KM plots also showed that the risks of all-cause and cardiovascular mortality were significantly elevated in patients with higher ePWV.

**Conclusions:**

ePWV had a close association with all-cause and cardiovascular mortality risks in patients with diabetes.

## Introduction

Arterial stiffness is an important cardiovascular risk factor and strongly associated with increased mortality (1, 2). Arterial stiffness can be assessed by carotid-femoral pulse wave velocity (cf-PWV), which is now listed in the guidelines for detecting organ damage caused by hypertension (3). However, cf-PWV is not widely used in clinical practice due to its measurement needs experienced staff and specialized equipment. Estimated pulse wave velocity (ePWV) is a novel index calculated based on mean blood pressure (MBP) and age (4–6). Although ePWV cannot substitute cf-PWV, it has been validated that ePWV reliably reflects the degree of arterial stiffness (7–9). Moreover, studies already demonstrated that ePWV had predictive value for cardiovascular risks (10, 11). Nevertheless, whether ePWV is associated with all-cause mortality and cardiovascular mortality in patients with diabetes remains exclusive.

Vascular remodeling is thought to be an important feature of human aging, loss of arterial elasticity is common as people age (12, 13). But at the individual level, vascular remodeling is actually a dynamic process, influenced by life style, genetic/epigenetic factors, and biological cues (14–16). Diabetes is an important risk factor for vascular remodeling, which can cause extracellular matrix changes, eventually leading to arterial remodeling and arterial stiffness (17, 18). It has been reported that arterial stiffness is greater in patients with diabetes than in individuals without diabetes (19, 20). Zhang et al*.* observed that reactive oxygen species were significantly elevated in the arteries of STZ-induced diabetic rats, and oxidative stress can lead to deposition of collagen and arterial fibrosis (21). Besides, arterial stiffness was shown to predict Alzheimer's disease, atrial fibrillation, and stroke (22–24). Therefore, more attention should be paid to arterial stiffness in patients with diabetes.

Although several studies have already investigated the association of ePWV and mortality in general population, to the best of our knowledge, the association between ePWV and mortality in the high-risk population of patients with diabetes remains exclusive. In the present study, based on a large multiracial population from National Health and Nutrition Examination Survey (NHANES), we investigated the association of ePWV with all-cause mortality and cardiovascular mortality in patients with diabetes.

## Methods

### Study population

NHANES is a continuous cross-sectional survey, conducted once every 2 years by the National Center for Health Statistics in the Centers for Disease Control and Prevention (25, 26). The method of “stratified multistage probability sampling” was adopted to screen out representative participants in NHANES survey (26). Detailed methods are described in the NHANES website (http://www.cdc.gov/nchs/nhanes.htm). All participants enrolled in NHANES provided written informed consent, and the whole procedures were approved by the Institutional Review Board of the Centers for Disease Control and Prevention. An analysis of the ten consecutive NHANES circles (1999–2018) was performed in the present study. Participants meeting one or more of the following criteria were considered to have diabetes: (1) self-reported diabetes; (2) individuals with prescribed antidiabetic medications; (3) fasting glucose ≥7.0 mmol/L or plasma HbA1c ≥ 6.5% (27). Among a total of 101,316 participants from NHANES survey, 9,233 individuals were diagnosed as diabetes. After excluding 317 participants younger than 18 years old or pregnant, a total of 8,916 adults with diabetes were enrolled in the present study.

### ePWV measurement

The blood pressure was recorded by a trained examiner after participants resting quietly in a seated position for 5 min according to the protocol of blood pressure measurement released by the American Heart Association (28). The average systolic blood pressure (SBP) and diastolic blood pressure (DBP) of three consecutive measurements was obtained and reported. Same as previous published studies (29, 30), MBP was calculated as algorithm (1):

(1) MBP=DBP+[0.4×(SBP−DBP)]

ePWV was calculated based on the algorithm (2):

(2) $$\begin{array}{rl} ePWV= & \;9.587-(0.402\times age)+[4.560\times 0.001\times (age^{2})]\\ & -[2.621\times 0.00001\times (age^{2})\times MBP]\\ & +(3.176\times 0.001\times age\times MBP)\\ & -(1.832\times 0.01\times MBP) \end{array}$$

### Determination of mortality outcomes

Participants enrolled in this study were linked to the National Death Index (NDI) database (https://www.cdc.gov/nchs/data-linkage/mortality-public.htm) to obtain death certificate data and determine mortality status. Publicly accessible death data were adopted from the start of follow-up to December 31, 2019 (the last update date of NDI database). Mortality outcomes were determined according the International Statistical Classification of Diseases, 10th Revision (ICD-10) (31). In ICD-10, cardiovascular death is defined as a death caused by a disease or condition related to the circulatory system. ICD-10 codes for cardiovascular death include a range of codes related to heart failure (I50), cerebrovascular accidents or strokes (CVA) (I60–I69), and poisoning or adverse effects of agents affecting the cardiovascular system (T46).

### Covariates

Age, sex, race/ethnicity, education levels and family income were obtained from the demographic questionnaires of NHANES survey. Alcohol consumption, smoking status and status of other chronic diseases were adopted from the health questionnaires. Moreover, Charlson comorbidity index (CCI) was also calculated based on the heathy status of participants (32). Age (years) was used as a continuous variable. Sex was classified as male or female. Race/ethnicity was classified as Mexican American, non-Hispanic White, non-Hispanic Black, Mexican, and others. Body mass index (BMI) was measured and categorized into: <25.0, 25.0–29.9, and >29.9 kg/m^2^ according to the definition of obesity and overweight. Smoking status were categorized as never smoker, ever smoker, and current smoker as suggested by NHANES (33). Drinking status were categorized as nondrinker, low to moderate drinker, and heavy drinker as suggested by NHANES (34). Education levels were divided into three levels, less than high school, high school or equivalent, and college or above. After at least 8 h of an overnight fast, 5 ml blood samples were collected and used to examine the levels of serum insulin, blood glucose, HbA1c, total triglyceride (TG), total cholesterol (TC), high-density lipoprotein cholesterol (HDL-C), low-density lipoprotein cholesterol (LDL-C), C-reactive protein (CRP), NHANES website provided the detailed procedures in collecting blood biochemical measurements.

### Statistical methods

We followed the NHANES analytic and reporting guidance in the present study. To reduce bias induced by post-stratification, non-response, and oversampling, stratified multistage probability sampling was adopted in NHANES. According to the primary sampling unit, a specific sampling weight was assigned to each participant to ultimately ­­produce representative estimates in the nation-wide. All data was produced according to the finally 20-years survey weight in statistical analysis. Continuous variables were presented as the weighted mean [95% confidence interval (CI)], and categorical variables were represented as proportions (95% CI). Baseline characteristics and were compared using adjusted Wald test for continuous variables and Rao-Scott *χ*^2^ test for categorical variables. Restricted cubic spline was adopted to visualize the linear relationship between ePWV and all-cause mortality and cardiovascular mortality. The *p*-value of linearity in RCS analysis is determined by comparing the fit of a linear model to that of a more complex model that includes splines, using the likelihood ratio test to obtain a test statistic and its associated *p*-value. We adopted the currently most recommended method of selecting 3 knots for RCS analysis. Weighted multivariable Cox regression was used to further investigate the association of ePWV with mortality risks. Since ePWV is calculated based on age and blood pressure, we found significant multicollinearity between age, SBP, and ePWV in the first adjusted cox regression model (Supplementary Table S1). Therefore, model I was finally adjusted for sex, race/ethnicity, and study circle, and model II was finally adjusted for sex, race/ethnicity, study circle, education levels, smoking, drinking, hypertension, and CCI. Results of Cox regression were presented as Hazard ratios (HRs) and 95% CI. As the missing values were within an acceptable range of approximately 20%, we used multiple imputation to handle the missing data. A *P* value < 0.05 was considered significant. All statistical analyses were conducted using R software (version 4.1.6, R Foundation for Statistical Computing, Vienna, Austria).

## Results

### Characteristics of the study population

A total of 8,916 participants with diabetes from NHANES survey were included in this study, representing 27.4 million patients with diabetes in the US. The mean age of study population was 59.0 years, 51.3% of the participants were male. The weighted mean level of ePWV was 9.50 m/s, and the median ePWV was 9.73 m/s. As shown in Table 1, patients with higher ePWV (ePWV ≥ 9.73 m/s) were older (71.1 vs. 50.2 years), more often quit smoking, and more likely to suffer from hypertension (85.8% vs. 60.4%) and other cardiovascular diseases. The proportion of participants with CCI ≥ 2 was significant higher in participants with higher ePWV. The comparison of cardiometabolic markers between two groups was shown in Table 2.

**Table 1:** Baseline characteristics of study population.

	Overall (*n* = 8916)	ePWV < 9.73 m/s (*n* = 4458)	ePWV ≥ 9.73 m/s (*n* = 4458)	*p* value
Age, years	59.0 (58.5, 59.5)	71.1 (70.7, 71.4)	50.2 (49.7, 50.8)	<0.001[Table-fn table-fn3]
Male, %	51.3 (48.3, 54.4)	50.2 (48.0, 52.5)	52.1 (50.0, 54.3)	0.2
Race/ethnicity				<0.001[Table-fn table-fn3]
White	61.9 (56.8, 67.0)	68.2 (65.2, 71.1)	57.4 (54.2, 60.6)	
Black	14.6 (13.1, 16.1)	14.2 (12.2, 16.2)	14.9 (13.0, 16.8)	
Mexican	9.2 (7.7, 10.7)	6.3 (4.9, 7.8)	11.3 (9.4, 13.2)	
Others	14.3 (12.5, 16.0)	11.3 (9.8, 12.8)	16.4 (14.3, 18.6)	
BMI, kg/m^2^				<0.001[Table-fn table-fn3]
Normal weight (<25.0)	12.1 (11.0, 13.2)	14.9 (13.6, 16.2)	10.6 (9.3, 12.0)	
Over weight (25.0–29.9)	27.3 (25.3, 29.3)	33.3 (31.6, 35.0)	24.3 (21.9, 26.6)	
Obesity (≥ 30.0)	58.0 (54.7, 61.3)	51.8 (49.8, 53.7)	65.1 (62.6, 67.7)	
Drinking status				<0.001[Table-fn table-fn3]
Nondrinker	37.4 (34.5, 40.2)	44.0 (41.5, 46.4)	32.6 (30.1, 35.0)	
Low to moderate drinker	42.0 (39.2, 44.9)	41.0 (38.3, 43.7)	42.8 (40.4, 45.3)	
Heavy drinker	11.9 (10.7, 13.1)	4.9 (3.8, 5.9)	17.0 (15.3, 18.6)	
Not recorded	8.7 (7.8, 9.6)	10.2 (8.7, 11.6)	7.6 (6.6, 8.7)	
Smoking status				<0.001[Table-fn table-fn3]
Never smoker	48.6 (46.0, 51.3)	49.0 (46.7, 51.4)	48.4 (46.1, 50.6)	
Ever smoker	34.1 (31.7, 36.6)	41.9 (39.8, 44.0)	28.5 (26.4, 30.7)	
Current smoker	16.7 (15.2, 18.2)	9.0 (7.9, 10.1)	22.3 (20.4, 24.1)	
Not recorded	0.5 (0.3, 0.7)	0.0 (0.0, 0.1)	0.8 (0.4, 1.2)	
Education levels				<0.001[Table-fn table-fn3]
Less than high school	11.3 (10.1, 12.4)	14.8 (13.2, 16.5)	8.7 (7.5, 9.9)	
High school or equivalent	40.8 (37.8, 43.9)	40.8 (38.6, 43.1)	40.8 (38.5, 43.2)	
College or above	47.8 (45.0, 50.5)	44.1 (41.5, 46.6)	50.4 (48.1, 52.7)	
Not recorded	0.1 (0.0, 0.2)	0.3 (0.0, 0.5)	0.0 (0.0, 0.1)	
Family income				<0.001[Table-fn table-fn3]
<2,000$	23.2 (21.5, 24.9)	26.9 (24.9, 28.9)	20.5 (18.5, 22.5)	
≥2,000$	72.5 (68.4, 76.5)	68.1 (66.0, 70.3)	75.6 (73.4, 77.8)	
Not recorded	4.4 (3.4, 5.3)	5.0 (4.0, 6.0)	3.9 (2.5, 5.3)	
SBP, mmHg	130.3 (129.6, 131.0)	141.2 (140.3, 142.1)	122.4 (121.8, 123.1)	<0.001[Table-fn table-fn3]
DBP, mmHg	70.1 (69.5, 70.6)	69.0 (68.3, 69.6)	70.8 (70.2, 71.5)	<0.001[Table-fn table-fn3]
Hypertension, %	71.0 (67.3, 74.8)	85.8 (84.3, 87.2)	60.4 (57.9, 62.9)	<0.001[Table-fn table-fn3]
Hyperlipidemia, %	86.8 (82.4, 91.3)	87.4 (86.0, 88.9)	86.4 (85.0, 87.8)	0.3
CVD, %	24.4 (22.5, 26.3)	35.8 (33.6, 37.9)	16.3 (14.6, 18.0)	<0.001[Table-fn table-fn3]
CCI				<0.001[Table-fn table-fn3]
>2	39.8 (37.2, 42.4)	47.2 (45.1, 49.3)	34.5 (32.2, 36.8)	
0	6.9 (6.2, 7.7)	4.1 (3.4, 4.9)	9.0 (7.8, 10.1)	
1	33.4 (31.2, 35.6)	25.7 (23.8, 27.6)	39.1 (36.8, 41.3)	
2	19.8 (18.2, 21.3)	22.9 (21.1, 24.8)	17.5 (15.7, 19.3)	

Continuous variables are presented as the mean and 95% confidence interval, category variables are presented as the proportion and 95% confidence interval.

ePWV, estimated pulse wave velocity; BMI, body mass index; SBP, systolic blood pressure; DBP, diastolic blood pressure; CVD, cardiovascular disease; CCI, charlson comorbidity index.

* *P* value < 0.001.

**Table 2:** Cardiometabolic markers of study population.

Variables	Overall (*n* = 8916)	ePWV <9.73 m/s (*n* = 4458)	ePWV ≥ 9.73 m/s (*n* = 4,458)	*p* value
Insulin, pmol/L	132.8 (126.2, 139.4)	140.1 (130.4, 149.8)	122.6 (113.5, 131.7)	0.01
Glucose, mmol/L	8.5 (8.3, 8.7)	8.7 (8.5, 9.0)	8.1 (8.0, 8.3)	<0.001[Table-fn table-fn6]
HOMA-IR	8.7 (8.2, 9.2)	9.3 (8.5, 10.1)	7.9 (7.3, 8.5)	0.01
HbA1c, %	7.2 (7.1, 7.2)	7.3 (7.2, 7.4)	6.9 (6.9, 7.0)	<0.001[Table-fn table-fn6]
TG, mmol/L	2.0 (1.9, 2.1)	2.2 (2.0, 2.4)	1.8 (1.7, 1.8)	<0.001[Table-fn table-fn6]
TC, mmol/L	4.9 (4.9, 5.0)	5.0 (5.0, 5.1)	4.8 (4.8, 4.9)	<0.001[Table-fn table-fn6]
HDL-C, mmol/L	1.2 (1.2, 1.2)	1.2 (1.2, 1.2)	1.3 (1.3, 1.3)	<0.001[Table-fn table-fn6]
LDL-C, mmol/L	2.8 (2.7, 2.8)	2.9 (2.8, 2.9)	2.7 (2.6, 2.7)	<0.001[Table-fn table-fn6]
CRP, mg/dl	0.6 (0.6, 0.6)	0.6 (0.6, 0.7)	0.5 (0.5, 0.6)	0.01

Variables are presented as the mean and 95% confidence interval.

ePWV, estimated pulse wave velocity; HOMA-IR, homeostasis model assessment-insulin resistance; TG, total triglyceride; TC, total cholesterol; HDL-C, high-density lipoprotein cholesterol; LDL-C, low-density lipoprotein cholesterol; CRP, C-reactive protein.

* *P* value < 0.001.

### Associations of ePWV with all-cause mortality and cardiovascular mortality

**Figure 1:**
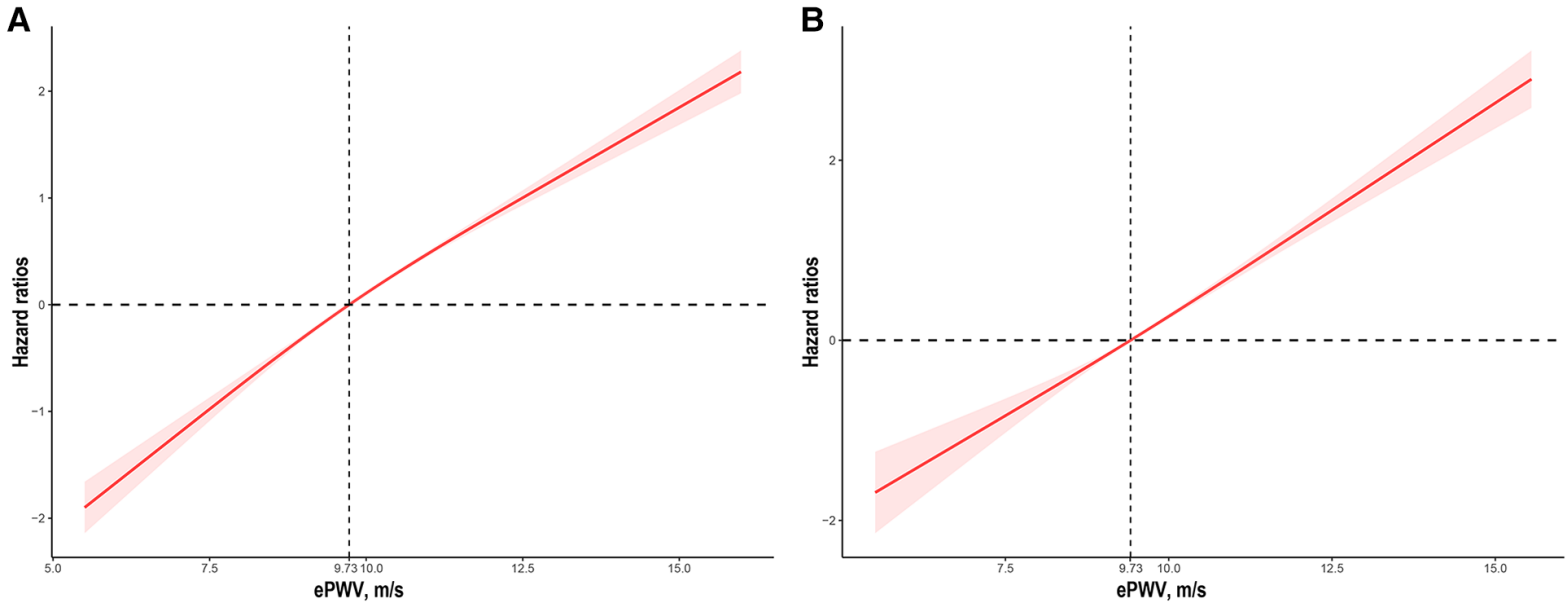
The association of ePWV with all-cause (**A**) and cardiovascular mortality (**B**) among participants with diabetes visualized by the restricted cubic spline. HRs were adjusted for sex, race/ethnicity, study circles, education levels, smoking, drinking, hypertension, and CCI. Both *p* for linearity <0.001. ePWV, estimated pulse wave velocity; CCI, Charlson comorbidity index.

**Figure 2:**
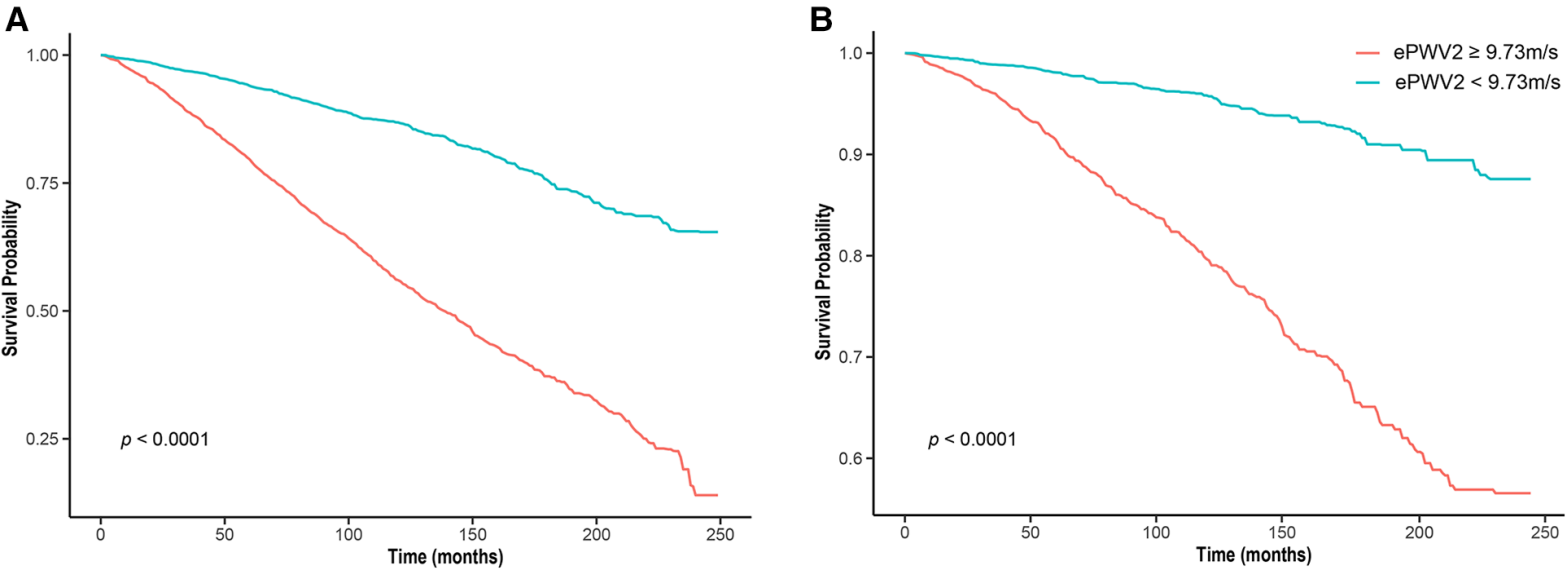
Kaplan–Meier survival curve of patients with lower (< 9.73m/s) ePWV and higher (≥ 9.73m/s) ePWV. ePWV, estimated pulse wave velocity (**A**) all cause survival; (**B**) cardiovascular cause survival.

**Figure 3:**
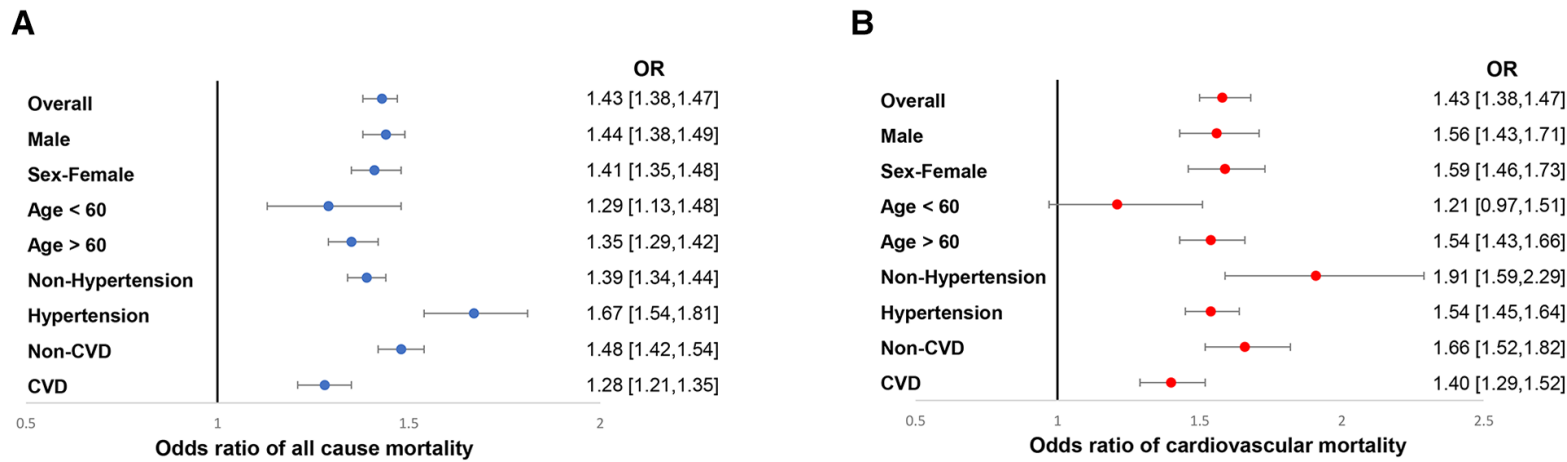
Subgroup analysis of the association between ePWV with all-cause (**A**) and cardiovascular mortality (**B**) among participants with diabetes. HRs were adjusted for sex, race/ethnicity, study circles, education levels, smoking, drinking, hypertension, and CCI. ePWV, estimated pulse wave velocity; CCI, Charlson comorbidity index.

The median follow-up time was 10.0 (95% CI: 9.8–10.2) years. During that time, 2,407 (27.0%) of 8,916 patients with diabetes died, including 684 (7.7%) cardiovascular deaths. Weighted all-cause mortality (1,000 person-years) of the study population was 33.6 (95% CI: 32.8–33.9), and weighted cardiovascular mortality was 5.6 (95% CI: 5.2–5.8). Results of restricted cubic spline showed that ePWV positively correlated with all-cause mortality and cardiovascular mortality, the linear relationship was visualized in Figure 1. Weighted Kaplan-Meier (KM) plots also showed the risks of all-cause mortality (Figure 2A) and cardiovascular mortality (Figure 2B) were significantly elevated in patients with higher ePWV. We also adopted weighted multivariable Cox regression analyses to investigate the association between ePWV and mortality risk. In the non-adjusted model, we found that every 1 m/s increase in ePWV, there was a 46% increased risk of all-cause mortality (HR: 1.46, 95% CI: 1.42–1.51) and 59% increased risk of cardiovascular mortality (HR: 1.59, 95% CI: 1.50–1.68). After adjusting for confounding factors including sex, race/ethnicity, study circle, education levels, smoking, drinking, hypertension, and CCI, the relationship between ePWV and all-cause mortality (HR: 1.43, 95% CI: 1.38–1.47) and cardiovascular mortality (HR: 1.58, 95% CI: 1.50–1.68) were still significant (Table 3). Although covariates were adjusted, considering individuals with higher ePWV have a higher proportion of pre-existing cardiovascular disease due to the characteristics of ePWV that are highly associated with age. Subgroup analysis stratified by age, gender, presence of hypertension, and cardiovascular disease as you suggested. In patients with pre-existing cardiovascular disease and patients without pre-existing cardiovascular disease, ePWV both showed a favor prognostic value (Figure 3). Since ePWV is calculated based on age and blood pressure, we found significant multicollinearity between age, SBP, and ePWV. However, considering age and SBP are strongest risk factor for all-cause and cardiovascular mortality, we further included age and SBP in the model to determine whether ePWV is an independent risk factor for cardiovascular mortality. Of note, we found that ePWV is not an independent risk factor for all-cause mortality (HR: 1.08, 95% CI: 0.98–1.2, *p* = 0.12) and cardiovascular mortality (HR: 1.18, 95% CI: 0.98–1.42, *p* = 0.09) adjusting for age and SBP.

**Table 3:** Cox regression analysis on the association between ePWV and All-cause mortality and cardiovascular mortality.

	Non-adjusted model		Model I		Model II	
	HR [95% CI]	*P* value	HR [95% CI]	*P* value	HR [95% CI]	*P* value
All-cause mortality	1.46 [1.42, 1.51]	<0.001	1.47 [1.43,1.51]	<0.001	1.43 [1.38,1.47]	<0.001
Cardiovascular mortality	1.59 [1.50, 1.68]	<0.001	1.60 [1.52,1.70]	<0.001	1.58 [1.50, 1.68]	<0.001

HR per 1 m/s increase in ePWV with 95% CIs. Model I adjusted for sex, race/ethnicity, and study circle. Model II adjusted for sex, race/ethnicity, study circle, education levels, smoking, drinking, hypertension, and CCI.

HR, Hazard ratio; CI, confidence interval; ePWV, estimated pulse wave velocity; CCI, Charlson comorbidity index.

### Evaluation of predicting value of ePWV for all-cause mortality and cardiovascular mortality

**Figure 4:**
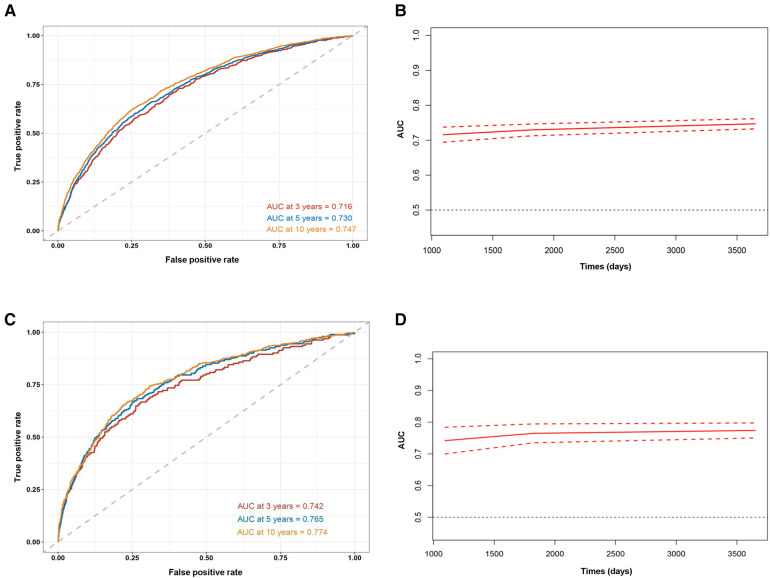
Time-dependent ROC curves **(A)** and time-dependent AUC values **(B)** of ePWV predicting all-cause mortality, and time-dependent ROC curves **(C)** and time-dependent AUC values **(D)** of ePWV predicting cardiovascular mortality.

We also carried out time-dependent ROC curve analysis to evaluate the prognostic value of ePWV for all-cause mortality and cardiovascular mortality. The results showed that ePWV had a good predicting value of all-cause mortality (Figures 4A,B) and cardiovascular mortality (Figures 4C,D) in short-term, medium-term, and long-term. We found that best cut-off value of ePWV for 3 years, 5 years, and 10 years all-cause mortality were 14.23, 9.95, and 12.45, respectively. The best cut-off value of ePWV for 3 years, 5 years, and 10 years cardiovascular mortality were 7.53, 9.17, and 12.63, respectively.

## Discussion

Concurring with aging, arterial stiffness is a type of inflammatory lesion of the artery, which can make the arterial wall thick, and make the lumen narrow (35). The most important causes of arterial stiffness are high blood pressure, hyperlipidemia and smoking. Moreover, obesity, diabetes, physical inactivity, and stress can all contribute to arterial stiffness (36). The manifestations of arterial stiffness are mainly determined by the degree of ischemia in the affected organs. Most of the patients with early arterial stiffness nearly have no clinical symptoms. However, numerous studies have already demonstrated that arterial stiffness can lead to increased cardiovascular risk (8, 29, 37). There is a lack of sensitive and specific laboratory diagnostic methods for early arterial stiffness. cf-PWV is considered as the gold standard method for diagnosing arterial stiffness. It is advisable to measure cf-PWV to determine whether there is arterial stiffness, as well as the location according to Doppler ultrasound. According to the guidelines, cf-PWV greater than 10 m/s indicates the occurrence of arterial stiffness (1). Nevertheless, as previously mentioned, cf-PWV is not widely used in current clinical practice due to its measurement requires specialized equipment. It is important to understand the status of arterial stiffness, especially in high-risk individuals such as patients with cardiovascular and cerebrovascular diseases. ePWV is a novel index calculated based age and MBP, which was firstly proposed in 2010 to estimate the degree of arterial stiffness (38). The use of ePWV has increased awareness of the dangers of aortic stiffness and helped physicians take advantage of it in clinical practice (10, 39, 40). In the present study, we adopted the previously published equation to calculate ePWV and applied it in a high-risk population with diabetes.

The 2018 European Society of Hypertension/European Society of Cardiology (ESH/ESC) guideline for the management of hypertension recommend measuring PWV as a marker of target organ damage in order to improve cardiovascular risk prediction. In recent years, the clinical value of ePWV has been extensively investigated (3). Vishram et al*.* conducted a prospective study based on a large low-risk European cohort and demonstrated that ePWV can predict cardiovascular outcomes in the general population (11). In a NHANES analysis published in *J Am Coll Cardiol*, investigators also found increased ePWV has a strong association with cardiovascular mortality and all-cause mortality risks in general population (37). It is interesting to note that Heffernan et al*.* performed another study with NHANES data and showed ePWV even had a predictive value for the residual-specific mortality (41). However, the exploration of roles of ePWV in high-risk population is still needed. Arterial stiffness is an important contributor to hypertension and hypertension can further promote arterial stiffness, resulting in a vicious cycle. Vlachopoulos et al*.* investigated roles of ePWV in high-risk individuals from the SPRINT trial. After 3.26 years follow-up, the researchers found that ePWV was associated with all-cause mortality and cardiovascular events in patients with hypertension (29). Study of Hametner et al*.* showed that ePWV was not only associated with mortality, but also risks of unplanned revascularization, stroke, and myocardial infarction among patients with suspected coronary artery disease undergoing invasive angiography (42). Our study paid attention to another high-risk population with diabetes. Compared with the study based on SPRINT cohort focusing on roles of ePWV in patients with hypertension, our study (i) has substantially longer follow-up time (median 10 years vs. median 3.26 years), (ii) recruited a similar number of participants (8,916 vs. 9,361 individuals), (iii) adjusted for more extensive confounding factors, such as CCI.

Diabetes is an important cause of arterial stiffness, and it is very necessary to pay attention to ePWV of patients with diabetes. Clinical studies have shown that patients with diabetes are more likely to develop arterial stiffness and had a higher cf-PWV than people without diabetes (43–45). The molecular mechanism of arterial stiffness caused by diabetes has also been widely investigated. Zhang et al*.* found that diabetes can activate MMP2, MMP9, and TGF*β*1/Smad2/3 pathways, leading to elevated blood pressure and arterial stiffness (46). Glucose fluctuations often occurs in patients with diabetes after meals. Another study focused on the impact of glucose fluctuations on arterial stiffness showed that glucose fluctuations aggravated aortic fibrosis by promoting oxidative stress and activating Runx2 (21). Arterial stiffness can be used as an important prognostic indicator for patients with diabetes. Therefore, we conduct this study to evaluate the prognostic value of ePWV in a large high-risk population with diabetes from NHANES survey. In the present study, the increment of ePWV is closely associated with increased all-cause and cardiovascular mortality. Our results in this high-risk population (patients with diabetes) add to the existed findings from previous studies in low- and high-risk populations (general population and patients with hypertension). Therefore, controlling arterial stiffness is very important especially for patients with diabetes.

There are several strengths of our study. First, it was adequate to provide reliable conclusion and statistical power considering the large-scale sample size included and long follow-up duration; second, the participants included in this study were from NHANES survey, reducing selection bias caused by selective inclusion of specific hospitals and health insurance system; third, follow-up and mortality information were directly obtained from NDI database, it was collected by high-quality nationwide registers, and patients were followed until death. However, several limitations of this study should be mentioned. First, cf-PWV is not available in NHANES database, therefore, the compassion of ePWV and cf-PWV on the prognostic potential needs to be further explored; second, there may exist experienced subjective bias due to the self-reported covariates from NHANES database; third, whether the conclusion in the present study based on US participants could be applicable to other populations need to be further explored in the future work.

## Conclusion

We analyzed 8,916 adult patients with diabetes from NHANES survey and found that ePWV is associated with all-cause and cardiovascular mortality risks during a long-term follow-up. Therefore, earlier prevention against arterial stiffness is important for patients with diabetes.

## Data Availability

The datasets presented in this study can be found in online repositories. The names of the repository/repositories and accession number(s) can be found in the article/**supplementary material**.
