# Supplementary material for: Estimated pulse wave velocity is associated with all-cause mortality and cardiovascular mortality among adults with diabetes
Source: Front Cardiovasc Med. 2023 Apr 17;10:1157163. doi: 10.3389/fcvm.2023.1157163 (PMC10150383; doi:10.3389/fcvm.2023.1157163)
Supplement: Supplementary file 1 [file Table1.docx]

**Table S1. Evaluation of the multicollinearity of the Cox regressive mode II.**

| **Variables** | **ePWV** | **Age** | **Sex** | **Race** | **Study circles** | **Education levels** | **BMI** | **Smoking** | **Drinking** | **SBP** | **Hypertension** | **CCI** |
| --- | --- | --- | --- | --- | --- | --- | --- | --- | --- | --- | --- | --- |
| **VIF value** | 10.893 | 7.596 | 1.175 | 1.437 | 1.150 | 1.393 | 1.171 | 1.302 | 1.257 | 3.491 | 1.180 | 1.087 |

VIF, variance inflation factor; ePWV, estimated pulse wave velocity; BMI, body mass index; SBP, systolic blood pressure; CCI, charlson comorbidity index.
